# Senkyunolide H reverses depression-induced breast cancer progression by regulating CXCR2

**DOI:** 10.1007/s13659-025-00543-6

**Published:** 2025-08-21

**Authors:** Yingchao Wu, Jiaqi Cui, Liushan Chen, Jieting Chen, Junfeng Huang, Congwen Yang, Yuqi Liang, Qianjun Chen, Qian Zuo

**Affiliations:** 1 Chinese Medicine Guangdong Laboratory, Hengqin, 519031 Guangdong China; 2 State Key Laboratory of Traditional Chinese Medicine Syndrome, The Second Affiliated Hospital of Guangzhou University of Chinese Medicine, Guangzhou, 510006 Guangdong China; 3 Department of Breast, Guangdong Provincial Hospital of Chinese Medicine, 111 Dade Road, Guangzhou, 510006 Guangdong China; 4 The Second Clinical College of Guangzhou, University of Chinese Medicine, Guangzhou, 510006 Guangdong China; 5 College of Traditional Chinese Medicine, Jinan University, Guangzhou, 510405 Guangdong China; 6 The First Clinical College of Guangzhou, University of Chinese Medicine, Guangzhou, 510405 Guangdong China

**Keywords:** Breast cancer, Depression, IL-8, CXCR2, Senkyunolide H

## Abstract

**Background:**

Depression promotes breast cancer progression. Given the lack of specific targets for depression-associated breast cancer, there are currently no therapeutic drugs for this type of breast cancer.

**Methods:**

Transcriptomic analysis was conducted to identify and functionally annotate genes with differential expression in breast cancer patients exhibiting depressive symptoms. Subsequently, Mendelian randomization was employed to investigate the causal associations between these pivotal genes and breast cancer, thereby validating their potential roles as therapeutic targets. Furthermore, molecular docking techniques were utilized to screen for candidate compounds that may exert therapeutic effects on depression-associated breast cancer. The efficacy of the selected compounds was further assessed using both in vitro cellular experiments and in vivo animal models.

**Results:**

We identified IL-8 as a key gene involved in depression-mediated breast cancer progression using transcriptomics. Mendelian randomized analysis suggested that high IL-8 expression promoted breast cancer progression. Further studies demonstrated that IL-8 mediated the breast cancer-promoting effect of depression through the receptor CXCR2. Evidence from both in vitro and in vivo experiments indicates that senkyunolide H may exert its therapeutic effect by regulating CXCR2, thereby counteracting the protumor effects associated with depression in breast cancer.

**Conclusion:**

Depression activates CXCR2-mediated breast cancer cell proliferation through IL-8, and senkyunolide H regulates CXCR2 and inhibits its ability to block the cancer-promoting effects of depression, ultimately inhibiting the growth of breast cancer in the context of depression.

**Graphical Abstract:**

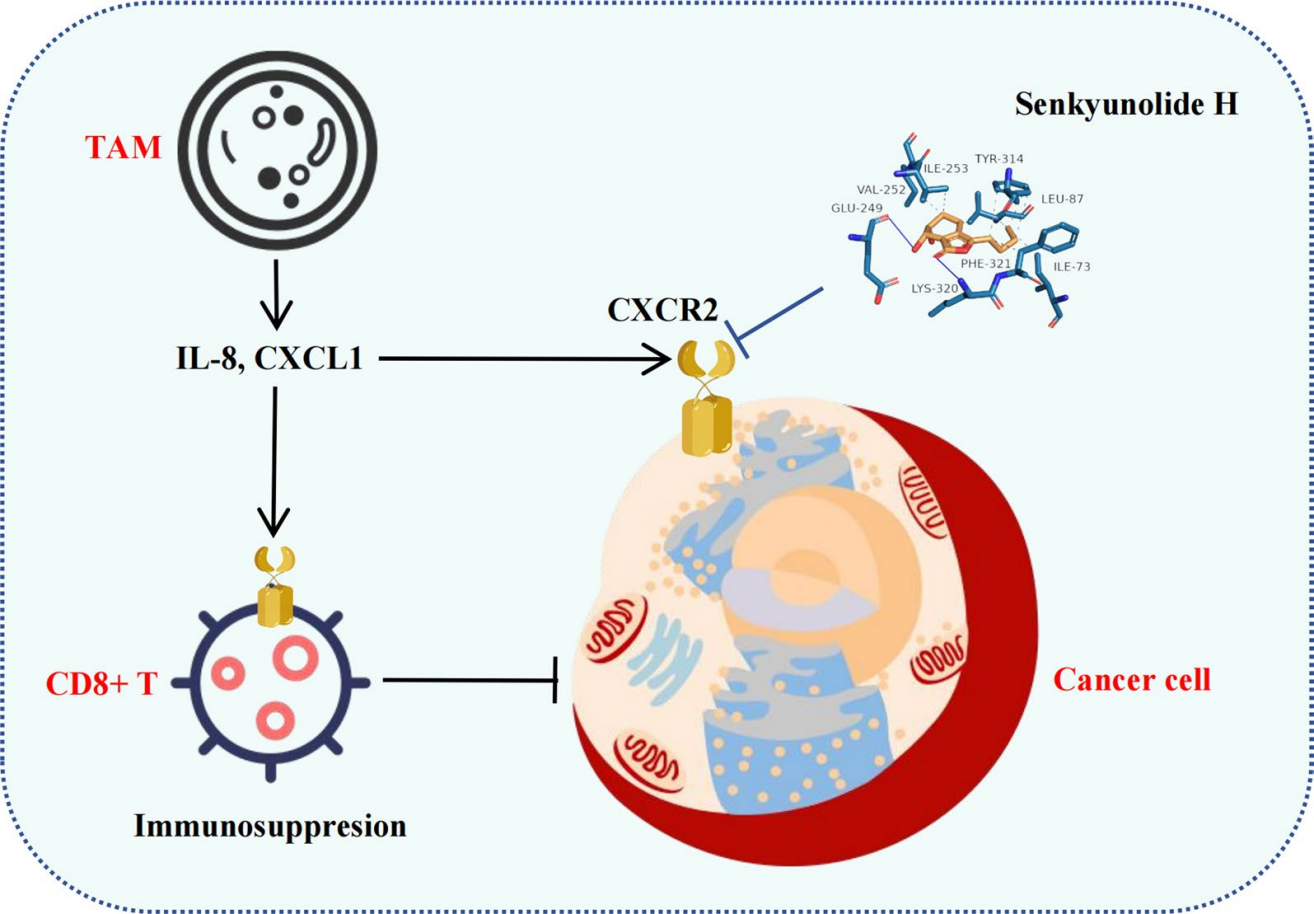

## Introduction

The latest survey estimates that 125.3 million people worldwide suffer from mental disorders such as depression [1]. On the other hand, the annual number of new cases of breast cancer is also increasing [2]. In this context, the number of breast cancer patients with depression is increasing. Similarly, increasing amounts of epidemiological evidence have demonstrated that negative emotional states, particularly depressive and anxiety disorders, are associated with elevated risks of both breast cancer incidence and mortality [3]. Research has indicated that emotional responses (e.g., anxiety and depression) under chronic stress conditions activate the hypothalamic‒pituitary‒adrenal (HPA) axis and the sympathetic nervous system within the central nervous system. This activation mediates the release of neurochemicals such as glucocorticoids and catecholamines, thereby inducing remodeling of peripheral cancer cells and the tumor immune microenvironment [4, 5]. However, most of the previous studies on the effects of depression on breast cancer have focused on the self-regulatory mechanism of the HPA axis and the influence of the HPA axis on the downstream macrophenotype, and research on the downstream micromolecular mechanism is lacking. It has been reported that depression promotes tumor growth and metastasis by affecting TAM/CXCL1 in the tumor microenvironment, but the downstream receptors of CXCL1 (such as CXCR1/CXCR2) have not been investigated in these studies [6–9]. Thus, the key molecular nodes involved in the promotion of breast cancer by depression have not been identified. Thus, understanding the alterations in the molecular mechanisms within the breast cancer microenvironment during the progression of depression holds substantial importance for advancing integrated therapeutic strategies targeting breast cancer associated with depression.

Currently, there is a lack of effective clinical treatments that specifically target breast cancer complicated by depression. As a result, therapeutic approaches for these patients largely mirror those used for patients with breast cancer not complicated by depression, which contributes to notably poorer treatment outcomes than those of patients without comorbid depression [10, 11]. In contrast, traditional medicine has demonstrated promising efficacy in addressing depression in the context of breast cancer. Meta-analyses have indicated that modified Xiaoyao San, a classical traditional Chinese medicine formula, alleviates depressive symptoms in breast cancer patients while increasing the effectiveness of chemotherapy [12]. Additionally, fundamental research has shown that Jiawei Xiaoyao Wan may exert therapeutic effects by modulating neurotransmitter activity and inflammatory pathways in patients with breast cancer complicated by depression [13]. Senkyunolide H is derived from *Radix angelicae* in Xiaoyao San and is the key component of Xiaoyao San [14]. Studies have shown that senkyunolide H has antidepressant, anti-inflammatory and other effects [15–17], but its antitumor effect has not been reported. The malignant progression of breast cancer associated with depressive symptoms may be related to abnormal levels of inflammatory factors [18, 19]. Given the potential of senkyunolide H in the treatment of depression and the intervention of inflammatory cytokine pathways, in an effort to develop new therapeutic strategies for breast cancer, we conducted an in-depth evaluation of the efficacy of senkyunolide H in treating breast cancer associated with depressive symptoms.

In this study, we explored the molecular mechanisms underlying the promotion of breast cancer progression by depression through the analysis of clinical tissue samples from affected patients. Moreover, the therapeutic efficacy of senkyunolide H against breast cancer associated with depression, along with its underlying mechanisms of action, was validated through both in vitro and in vivo experimental models.

## Materials and methods

### Patient grouping and clinical tumor sample collection

Prior to surgery, breast cancer patients were stratified on the basis of their scores on the Hamilton Depression Scale (17-item version, HAMD-17) and the Hamilton Anxiety Scale (HAMA). Patients exhibiting scores ≥ 7 on both the HAMD-17 and HAMA were classified as having depressive symptoms. After the tumor tissue was removed during the operation, the tissue was rinsed with normal saline within 10 min in vitro to remove the blood and dirt on the surface of the tissue, and the tissue from nonstudy sites, such as adipose tissue, was removed. After the tissue was gently dried with sterile gauze, it was transferred into a prechilled cryogenic storage tube, rapidly frozen in liquid nitrogen, and subsequently stored at − 80 °C in an ultralow-temperature freezer. This experimental scheme was approved by the Ethics Committee of Guangdong Provincial Hospital of Chinese Medicine (Approval No. BF2019-120-04).

### RNA sequencing

The total RNA from each sample was individually isolated via TRIzol reagent (Invitrogen) following standard extraction protocols. The synthesis of cDNA libraries was carried out via the VAHTS Universal V6 RNA-seq Library Preparation Kit for Illumina (Vazyme, Inc.) in accordance with the manufacturer’s guidelines. Library integrity and quality were verified using the Agilent 2200 system, and high-throughput sequencing was subsequently conducted on the DNBSEQ-T7 platform employing a 150-base pair paired-end read approach. The raw reads were processed to remove adapter sequences and low-quality reads, yielding clean reads for downstream analysis. These clean reads were aligned to the human reference genome (Ensembl release 109) using STAR. Gene-level quantification was performed using HTSeq to obtain read counts, and transcript expression was normalized using the reads per kilobase of transcript per million mapped reads (RPKM) approach. Difference analysis and enrichment analysis were performed via OmicShare Tools [20]. Additionally, protein–protein interaction (PPI) networks were analyzed via the STRING database (https://cn.string-db.org), and survival curve and immune infiltration analyses were performed using the Xiantao Academic bioinformatics tool (www.xiantao.love).

### Immunofluorescence (IF)

The frozen tissue was embedded in optimal cutting temperature (OCT) solution and cut into 10 μm slices. The slices were immunostained with anti-IL-8 (1:200, Cat. #27095-1-AP, Proteintech, Hubei, China), anti-PCNA (1:200, Cat. #GB12010-100, Servicebio, Hubei, China), anti-Ki-67 (1:200, Cat. #28074-1-AP, Proteintech), anti-TROP2 (1:200, Cat. #GB111809-100, Servicebio), anti-F4/80 (1:200, Cat. #29414-1-AP, Proteintech), and anti-CD8 alpha (1:200, Cat. #GB15068-100, Servicebio) primary antibodies, followed by incubation with secondary antibodies. All the slices were subsequently stained with DAPI to label the nuclei. After three washes with TBST solution, the red or green fluorescence intensity was measured using a fluorescence microscope to quantify the protein content in the samples, and ImageJ was used for quantitative analysis.

### Western blotting (WB)

In brief, fresh tumor tissue samples or cell cultures were first lysed in RIPA buffer. The lysates were then centrifuged at 12,000×*g* for 15 min at 4 °C. The protein concentration in the resulting supernatant was quantified using an enhanced BCA protein assay kit (Cat. #P0010S; Beyotime Biotechnology, Shanghai, China). Equal amounts of protein were subsequently separated by SDS‒PAGE and transferred to PVDF membranes (Cat. #GVHP04700; Merck KGaA, Darmstadt, Germany). The membranes were blocked with TBST containing 5% skim milk for 1 h, followed by incubation with the appropriate primary antibodies, including anti-IL-8 (1:1000, Cat. #27095-1-AP, Proteintech), anti-CXCR2 (1:1000, Cat. #GB115557-100, Servicebio), anti-CXCR1 (1:1000, Cat. #GB11625-100, Servicebio), anti-CXCL1 (1:1000, Cat. #30322-1-AP, Proteintech), anti-PI3K (1:1000, Cat. #67121-1-Ig, Proteintech), anti-AKT (1:1000, Cat. #10176-2-AP, Proteintech), anti-p-AKT (1:1000, Cat. #66444-1-Ig, Proteintech), and anti-GAPDH (1:1000, Cat. #60004-1-Ig, Proteintech). Subsequently, the membranes were incubated with the appropriate secondary antibodies (1:8000) for 60 min at room temperature. After three washes with TBST solution, protein signals were detected using Beyotime's hypersensitive ECL kit (Cat. #P0018S; Beyotime Biotechnology). The density of the protein bands was then quantified and documented using a gel imaging system (ChemiDox™; Bio-Rad, USA), and ImageJ was used for quantitative analysis. GAPDH was used as the loading control.

### Mendelian randomization (MR) study design

Our experimental design follows a general two-sample MR design [21]. We utilized depression as an exposure factor. A diagnosis of breast cancer was considered the outcome of MR analysis. We sourced IL-8 gene eQTL data and breast cancer-related summary statistics (dataset ID: ukb-16890) from the GWAS repository maintained by the Integrative Epidemiology Unit (https://gwas.mrcieu.ac.uk/). All the statistical analyses were implemented in R (version 4.2.2) using the “TwoSampleMR” and “MR-PRESSO” packages. The key parameters of this study are set as follows: (1) genome-wide significance level at *P* < 5 × 10^−8^; (2) linkage disequilibrium clustering (r^2^ < 0.001, region size = 10,000 kb; and (3) F statistic > 10. Among the various analytical approaches, the inverse variance weighted (IVW) method serves as the primary estimator. A p value less than 0.05 was considered indicative of statistical significance.

### Lentivirus establishment and transfection

As described previously [22], transduction with lentiviral vectors expressing CXCR1-specific shRNA or CXCR2-specific shRNA was used to knock down CXCR1 or CXCR2 expression, and the NCs were added to 6-well plates seeded with Py230 breast cancer cells. Py230 cells were treated 48 h after transfection, and the extent of the shRNA-mediated knockdown of CXCR1 or CXCR2 expression was evaluated using WB analysis.

### Cell viability assessment

The cells were exposed to IL-8 (Cat. #K20584, Shanghai Yuanye Bio-Technology Co., Ltd., Shanghai, China) or senkyunolide H (Cat. #B21462, Shanghai Yuanye Bio-Technology Co., Ltd.) at the indicated concentrations for 48 h. The cells were then photographed under a microscope. Cell viability was evaluated via a methylthiazolyldiphenyl-tetrazolium bromide (MTT) assay (Cat. #V13154, Gibco, NY, USA). An automated microplate spectrophotometer (BioTek Instruments, Winooski, VT, USA) was used to determine the absorbance of each well at 490 nm.

### Colony formation assay

The cells were seeded in 6-well plates (200 cells/well in 2000 μL) and cultured for approximately 14 days. Colonies with more than 50 cells per colony were treated with IL-8 or senkyunolide H for 48 h. Subsequently, the medium was removed, and the cells were washed twice with PBS. After the cells were fixed with 4% paraformaldehyde, the colonies were stained with Giemsa for 5 min, and pictures were taken to record the results.

### Animal experiment and sample collection

Eighty female C57BL/6J mice, aged 8 weeks and weighing between 17 and 19 g, were obtained from Beijing HFK Bioscience Co., Ltd. (Beijing, China) for this study. In the figure, we present a schematic diagram of each part of the animal experiment. Chronic unpredictable mild stress (CUMS) group-associated mice were subjected to CUMS, including fasting, water fasting, tail clamping, restraint, diurnal reversal, tilting, wet bedding, and strobing, without the same stimuli on 2 consecutive days. After 4 weeks of CUMS, the mice were subcutaneously inoculated with Py230 breast cancer cells (1 × 10^6^ cells in 100 μL), and all the depression-modeling groups continued to receive CUMS. Senkyunolide H (7.5 mg/kg, administered intragastrically once a day for 28 days) was administered to the mice in the senkyunolide H-related group, and the same volume of saline was administered to the mice in the control group. The tumor volume was measured every 7 days, and the tumor size was calculated via the following formula: tumor volume = length × width2 × 1/2. After 28 days of treatment, the mice were anesthetized with pentobarbital (100 mg/kg, intraperitoneally) and euthanized by cervical dislocation. The tumor tissues were then collected for subsequent analysis. This experimental scheme was approved by the Laboratory Animal Ethics Committee of Guangdong Province Hospital of Chinese Medicine (Approval No. 2022079).

### Behavioral assays

#### Open field test

Each mouse was placed into a clean box (40 × 40 × 30 cm), and spontaneous locomotion was recorded using an infrared camera. A single test session lasted for 5 min under dim light. Movement track images and thermoimages were analyzed online. Data analysis was performed using EthoVision XT version 3.0 software (Noldus, Wageningen, Netherlands).

#### Elevated plus-maze

The experiment began by placing the mouse on the central platform (5 × 5 cm). During a 5-min test session, the animal was allowed to freely explore the arena, which included two open arms (30 × 5 × 0.5 cm) and two closed arms (30 × 5 × 15 cm) positioned opposite each other. The movement track images and thermoimages in each arm were analyzed using the EthoVision package.

### Molecular docking

The binding affinity between senkyunolide H and CXCR2 was validated by molecular docking. The 3D structure of senkyunolide H was obtained from the PubChem database. The CXCR2 protein (6lfl) structures acquired from the RCSB PDB database (https://www.rcsb.org) served as receptors. This structure was visualized separately using PyMOL, and subjected to dehydration, hydrogenation, and charge calculations using Mgtools (Version 1.5.6). Ligand and receptor structures were saved as pdbqt files. Subsequent molecular docking was performed utilizing AutoDock Vina (version 1.5.6), and the resulting higher-scoring conformations were visualized with PyMOL. The PLIP web tool (https://plip-tool.biotec.tu-dresden.de/plip-web/plip/index) facilitated the visualization of docking points.

### Cellular thermal shift assay (CETSA)

Py230 cells were lysed by freezing and thawing three times in liquid nitrogen. The cell lysates were then divided into two aliquots: one served as the control, and the other was treated with senkyunolide H (40 μM) for 30 min at room temperature. The lysates were subsequently heated to the desired temperatures (57–73 °C) and cooled on ice. Protein bands were detected by Western blotting.

### Flow cytometric analysis

To assess immune responses within the tumor microenvironment, flow cytometry was used to quantify CD8+ T cells. Additionally, the proportions of TNF-α-producing and IFN-γ-producing cells among the CD8+ T-cell subset were determined.

### Statistical analysis

All the data are expressed as the means ± standard deviations. Statistical analysis was performed using SPSS 13.0 (SPSS Inc., Chicago, IL, USA), and graphical visualization was performed via GraphPad Prism 9 (GraphPad Software, LLC, California, USA). Data between two groups were compared using one-way analysis of variance (ANOVA), whereas repeated measures data were analyzed using repeated-measures ANOVA. A p value of < 0.05 was considered statistically significant.

## Results

### IL-8 is highly expressed in the tumor tissue of breast cancer patients with depression

**Fig. 1:**
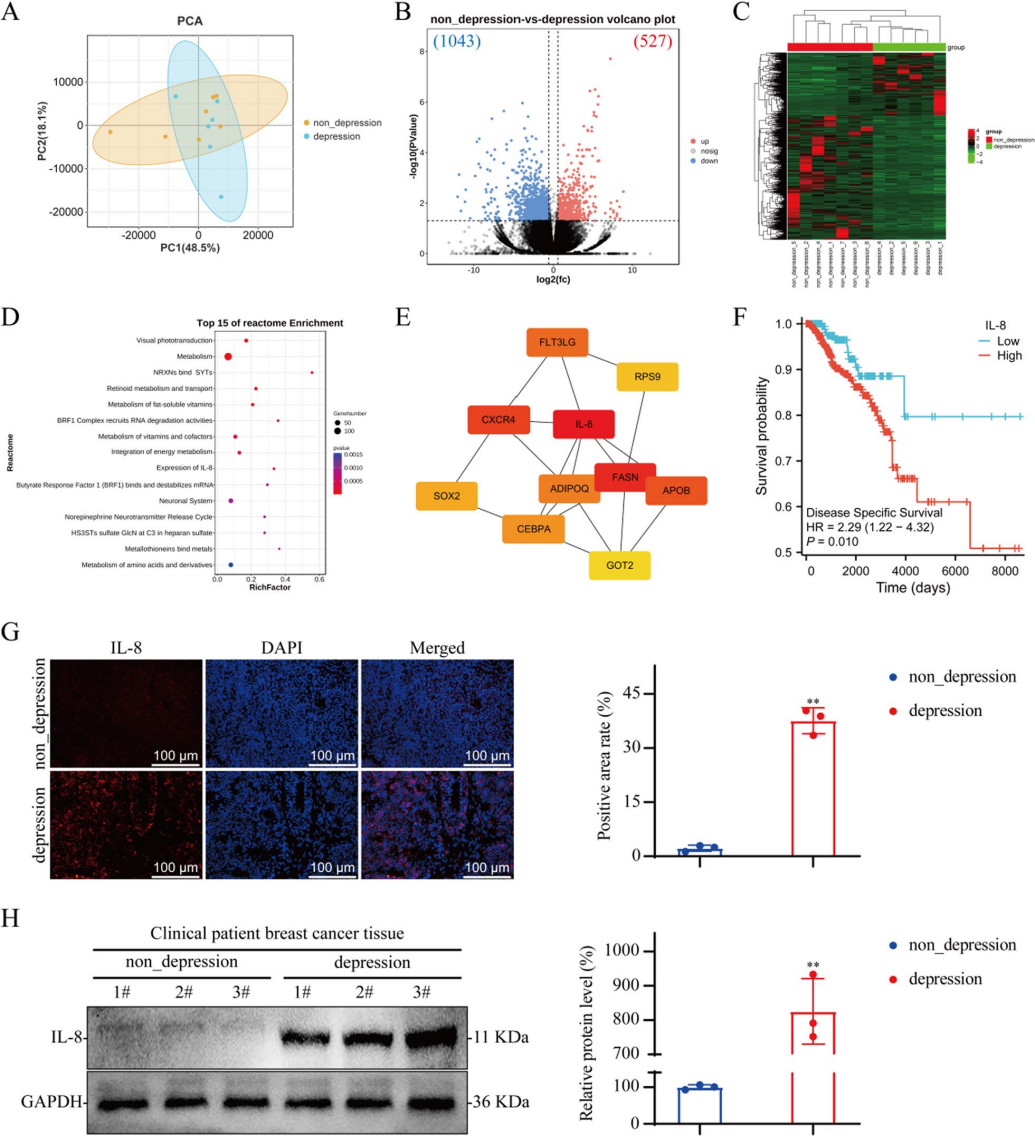
Analysis of protein expression in breast cancer tissues from nondepressed and depressed patients. **A** Principal component analysis map; nondepression group, n = 7; depression group, n = 6. **B** Volcano map. **C** Heatmap. **D** Reactome pathway enrichment of DEGs between the nondepression group and the depression group. **E** Protein‒protein interaction networks. **F** Kaplan‒Meier curves of IL-8 gene expression in breast cancer. **G** IF staining of tumor tissue from breast cancer patients; n = 3. **H** WB of tumor tissue from breast cancer patients; n = 3. The data are expressed as the means ± SDs; ^**^P < 0.01 compared with the nondepression group

Transcriptomic sequencing of breast cancer tissues from both nondepressed and depressed patients revealed that 527 genes were expressed at elevated levels in breast cancer tissues from depressed patients, and 1043 genes were expressed at lower levels in breast cancer tissues from depressed patients compared with those from nondepressed patients (Fig. 1A–C). The differentially expressed genes (DEGs) were subjected to Reactome pathway enrichment analysis, which revealed significant enrichment in pathways associated with IL-8 expression, the neuronal system, and the norepinephrine neurotransmitter release cycle (Fig. 1D). Further protein‒protein interaction analysis of these DEGs using degree and radiality ranking methods revealed that IL-8 ranked first among all the identified targets (Fig. 1E). Using the TCGA database, we analyzed the correlation between IL-8 expression in breast cancer tumor tissues and disease prognosis. The analysis revealed that patients with high IL-8 expression had a significantly reduced disease-specific survival probability (Fig. 1F). We performed IF and WB analyses of tumor tissue from both nondepressed and depressed patients and confirmed that IL-8 expression levels in breast cancer tissue from depressed patients was significantly increased (Fig. 1G and H).

### MR imaging suggests that IL-8 promotes breast cancer progression

**Fig. 2:**
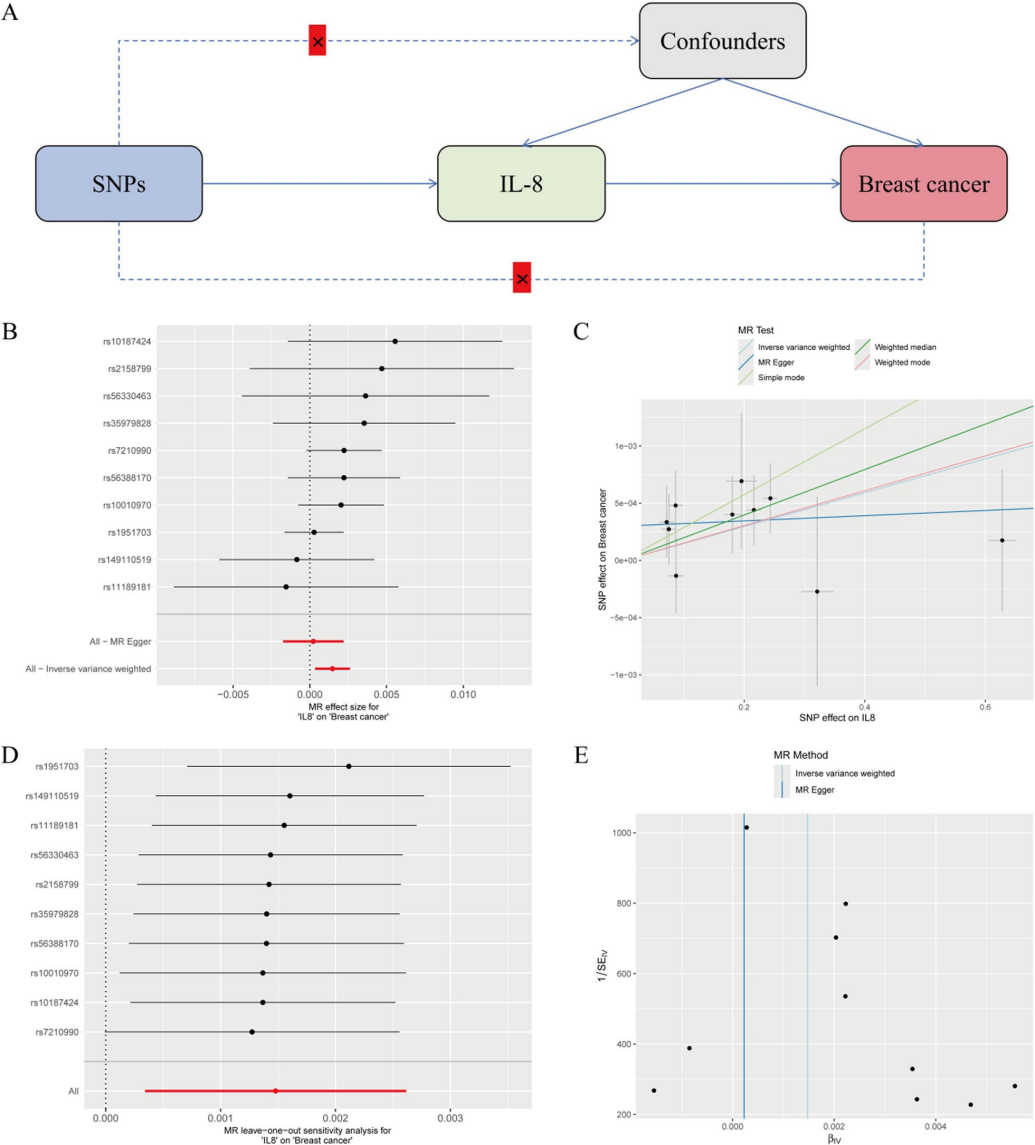
Mendelian randomized analysis of causality between IL-8 and breast cancer. **A** Diagram of the study design for MR. **B** A forest plot illustrating the causal effect of IL-8 SNPs on breast cancer incidence, with error bars representing the 95% confidence intervals (CIs). **C** Scatter plot of the causal SNP effect of IL-8 on the incidence of breast cancer. We plot each black point to represent each SNP on the exposure (horizontal axis) and the outcome (vertical axis), with error bars corresponding to each standard error (SEM). The slope of each line represents the combined estimate from the inverse variance weighted (light blue line), MR‒Egger (blue line), simple (light green line), weighted median (green line), and weighted (pink line) methods. **D** The leave-one-out analysis plot illustrates the causal effect of IL-8 on breast cancer, with error bars representing the 95% confidence intervals (CIs). **E** Funnel plot for the causal SNP effect of IL-8 on breast cancer incidence

To clarify the relationship between IL-8 expression and breast cancer progression, we performed two-sample MR analysis. The results of the MR analysis are presented in Fig. 2, including a schematic diagram of the research design (Fig. 2A), a forest plot (Fig. 2B), a scatter plot (Fig. 2C), a leave-one-out analysis plot (Fig. 2D), and a funnel plot (Fig. 2E). In this study, the correlation between exposure to IL-8 and breast cancer outcomes was analyzed using MR analysis. The results between IL-8 and breast cancer were statistically significant: *P* = 0.01 and 95% CI 1.000341–1.002619. No abnormal single nucleotide polymorphisms (SNPs) were identified in the leave-one-out analysis, reinforcing the idea that IL-8 could serve as a risk factor for breast cancer and may contribute to its development.

### Knocking down CXCR2 in tumor cells reverses the cancer-promoting effects of CUMS

**Fig. 3:**
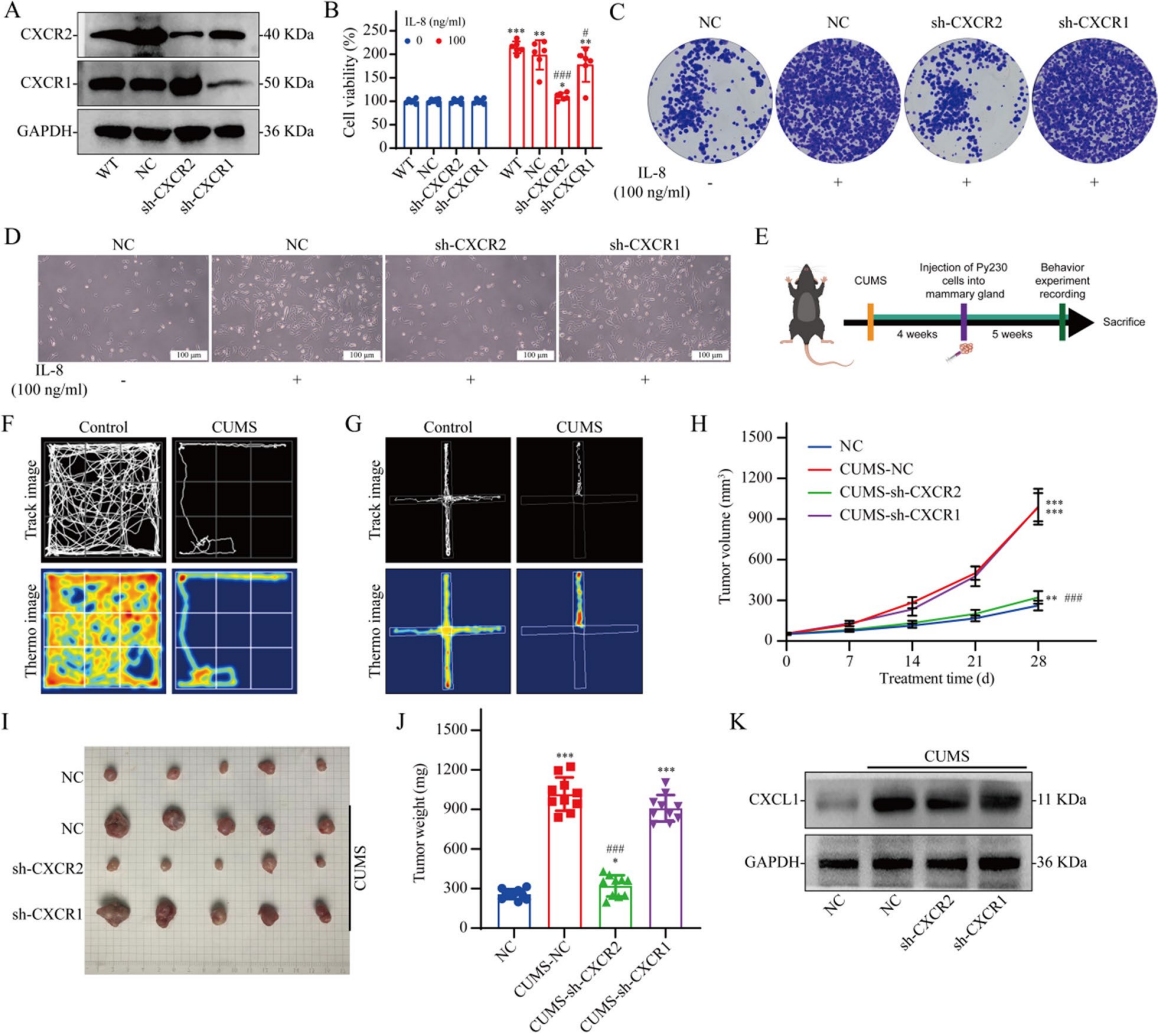
Effect of CXCR2 expression on the cancer-promoting effect of depression. **A** WB validation of the knockdown of the CXCR1 and CXCR2 proteins in Py230 cells. **B** Viability of Py230 cells in which CXCR1 or CXCR2 was knocked down 48 h after IL-8 treatment; n = 6. **C** Giemsa-stained colonies were visualized using an inverted microscope. **D** The number of cells was observed using an inverted microscope. **E** Flow chart of the in vivo experiments. **F** Open field test. **G** Forced swimming test. **H**–**J** Effect of a depressed mouse model on tumor growth in Py230 cells with CXCR1 or CXCR2 knockdown; n = 10. **K** WB of tumor tissue. The data are presented as the means ± SDs; ^*^P < 0.05, ^**^P < 0.01, ^***^P < 0.001 compared with the 0 ng/ml IL-8 group (in vitro) or NC group (in vivo); ^#^*P* < 0.05, ^###^*P* < 0.001 compared with the NC group (in vitro) or CUMS-NC group (in vivo)

The IL-8 receptors are CXCR1 and CXCR2. Therefore, we verified the effect of IL-8 on the growth of breast cancer cells using breast cancer cell lines with CXCR1 knockdown and CXCR2 knockdown (Fig. 3A). The results revealed that IL-8 significantly promoted breast cancer cell proliferation, whereas CXCR2 knockdown significantly reversed the promoting effect of IL-8 on the growth of breast cancer cells (Fig. 3B–D). We used in vivo experiments to verify the effect of CXCR2 on the cancer-promoting effects of depression (Fig. 3E). We first established a mouse model of depressive breast cancer (Fig. 3F and G). In vivo studies have shown that depression can significantly promote breast cancer growth. CXCR2 knockdown reverses the cancer-promoting effect of depression, whereas CXCR1 knockdown has no significant effect on the cancer-promoting effect of depression (Fig. 3H, I, J). We further analyzed IL-8 infiltration in tumor tissues from different groups using WB and found that the depression model significantly increased CXCL1 expression levels (the homolog of IL-8 in mice) in tumor tissues (Fig. 3K).

### Senkyunolide H regulates CXCR2 and reverses the cancer-promoting effects of IL-8

**Fig. 4:**
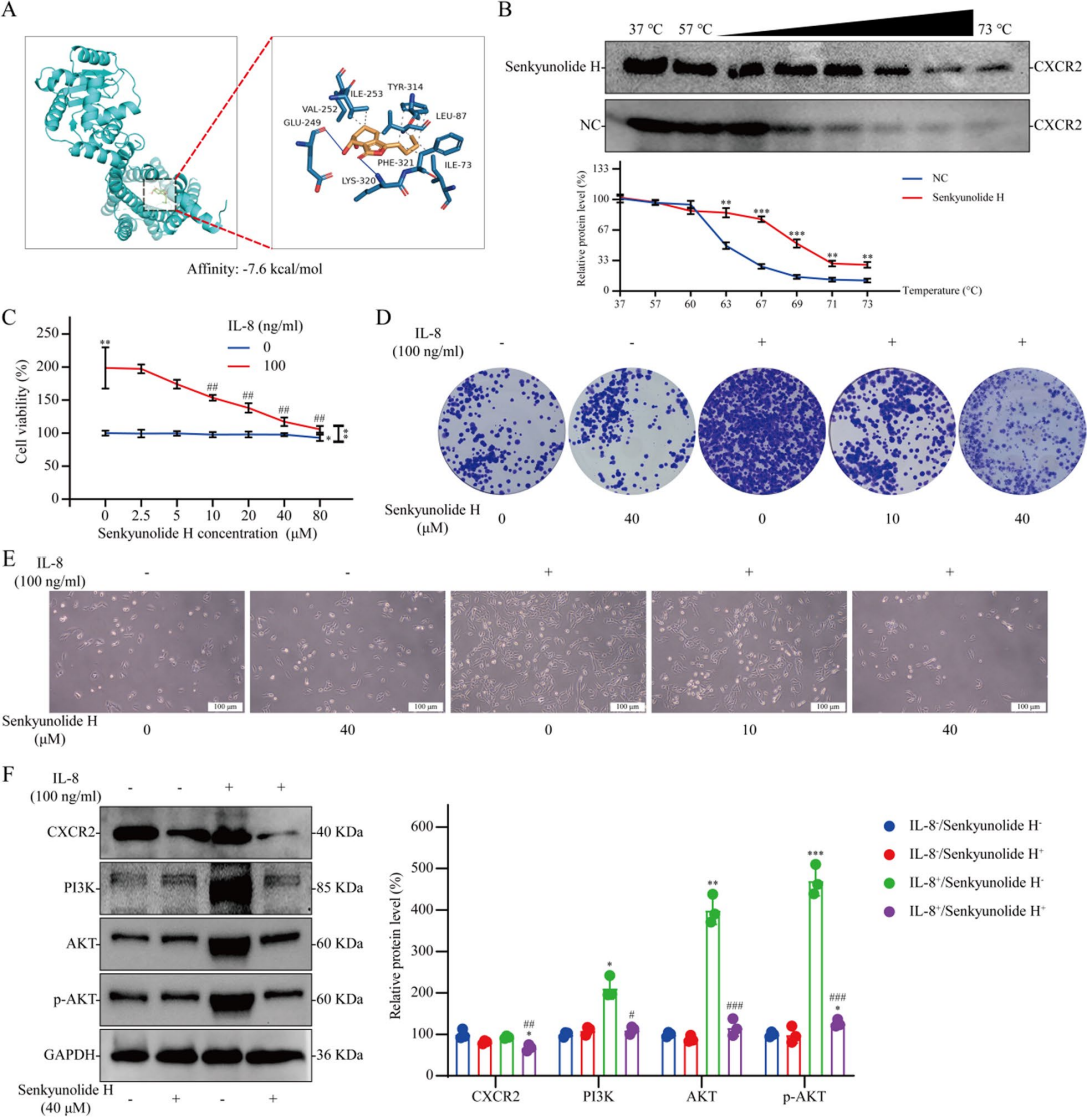
Effect of senkyunolide H on the tumor-promoting effect of IL-8 in vitro. **A** Molecular docking of senkyunolide H with the CXCR2 protein. **B** CETSA results; n = 3. **C** Viability of Py230 cells treated with or without IL-8 at different concentrations of senkyunolide H for 48 h; n = 6. **D** Giemsa-stained colonies were observed under an inverted microscope. **E** Effects of different IL-8 or senkyunolide H concentrations on cell growth. **F** Effects of IL-8 and/or senkyunolide H on protein expression in Py230 cells; n = 3. The data are presented as the means ± SDs; ^*^P < 0.05, ^**^P < 0.01, and ^***^P < 0.001 compared with the NC group or 0 ng/ml IL-8 group; ^#^*P* < 0.05, ^##^*P* < 0.01, and ^###^*P* < 0.001 compared with the 100 ng/ml IL-8 group

We discovered that senkyunolide H regulates CXCR2. The results of molecular docking revealed that senkyunolide H binds to the active pocket of the CXCR2 protein (Fig. 4A). To validate the interactions between senkyunolide H and CXCR2 in cells, we performed a CETSA. The results revealed that senkyunolide H treatment efficiently protected the CXCR2 protein from temperature-dependent degeneration, suggesting that senkyunolide H can directly regulate CXCR2 (Fig. 4B). We found that a low concentration of senkyunolide H had no significant inhibitory effect on breast cancer cells, but a low concentration of senkyunolide H significantly reversed the promoting effect of IL-8 on breast cancer cell proliferation (Fig. 4C and E). Clonal formation assays revealed that IL-8 markedly enhanced the clonal proliferation of Py230 breast cancer cells, an effect that was suppressed by senkyunolide H (Fig. 4D). WB analysis revealed that senkyunolide H CXCR2 protein levels. IL-8 significantly activated the PI3K/AKT signaling pathway in breast cancer cells, whereas senkyunolide H reversed PI3K/AKT signaling pathway activation. However, without IL-8 intervention, senkyunolide H had no significant effect on the PI3K/AKT signaling pathway in breast cancer cells (Fig. 4F).

### Senkyunolide H reverses the accelerated growth of breast cancer induced by CUMS

**Fig. 5:**
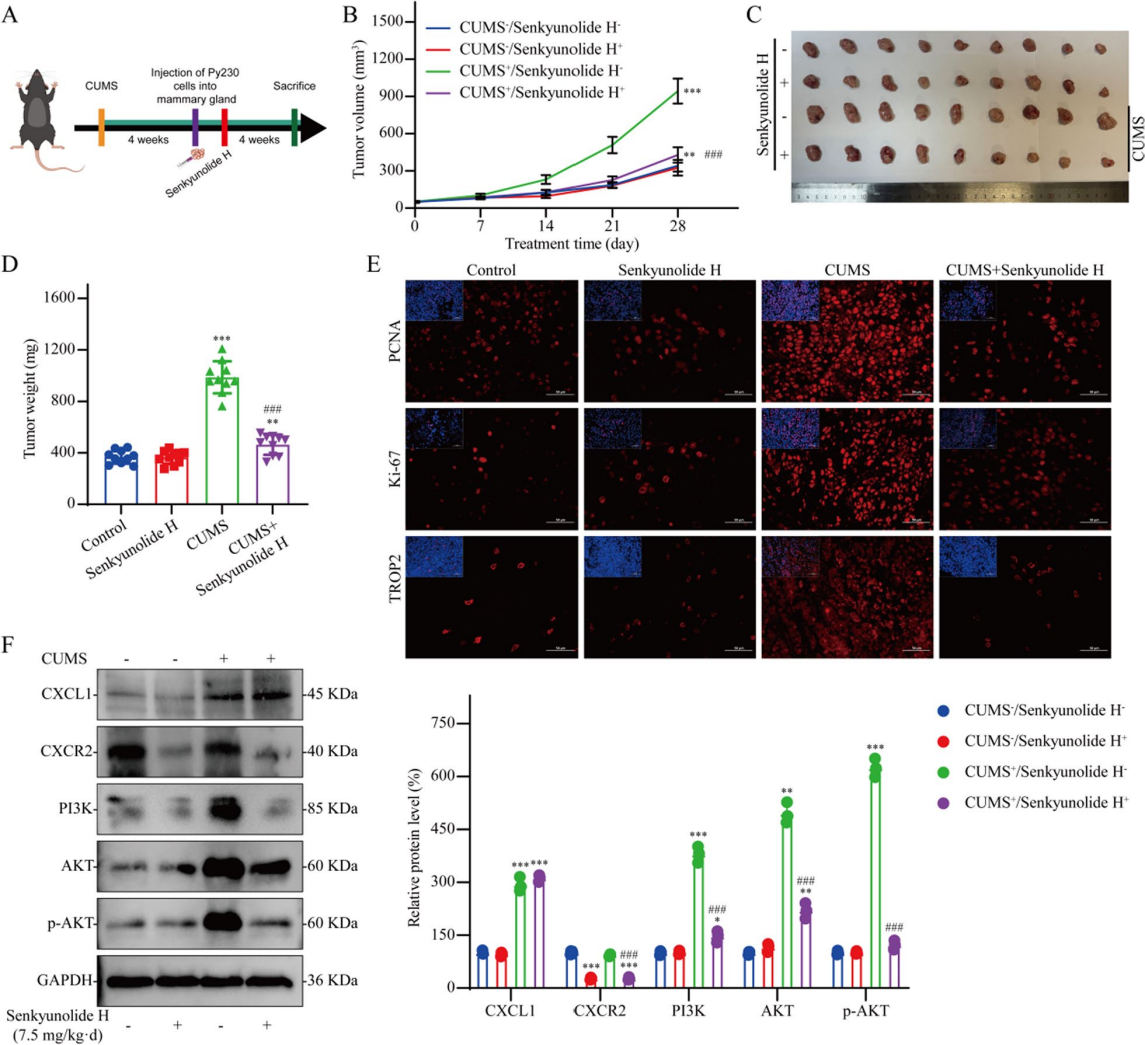
Effect of senkyunolide H on tumor promotion by CUMS in vivo. **A** Flow chart of the in vivo experiments. **B**–**D** Antitumor effects of senkyunolide H in vivo; n = 10. **E** IF staining of tumor tissue. **F** Western blot analysis of tumor samples; n = 3. The data are expressed as the means ± SDs; ^*^P < 0.05, ^**^P < 0.01, ^***^P < 0.001 in comparison with the CUMS−/senkyunolide H− group; ^###^*P* < 0.001 in comparison with the CUMS+/senkyunolide H− group

To verify the efficacy of senkyunolide H in the treatment of breast cancer patients with depression, we conducted further in vivo studies using a mouse model of breast cancer associated with depression (Fig. 5A). As shown in Fig. 5B–D, senkyunolide H had no significant anti-breast cancer effect on nondepressed mice with breast cancer, but senkyunolide H significantly reversed the protumor effect of CUMS. PCNA, Ki-67, and TROP2 are commonly used indicators for measuring the malignancy of breast cancer in clinical practice. CUMS significantly enhanced the malignant phenotype of breast cancer, but this change was effectively reversed by senkyunolide H (Fig. 5E). WB analysis revealed that senkyunolide H significantly inhibited CXCR2 expression. Although senkyunolide H did not reduce the infiltration level of tumor CXCL1, it significantly inhibited the activation of the PI3K/AKT signaling pathway induced by CUMS (Fig. 5F).

### Senkyunolide H remodels the antitumor immune microenvironment in the CUMS state

**Fig. 6:**
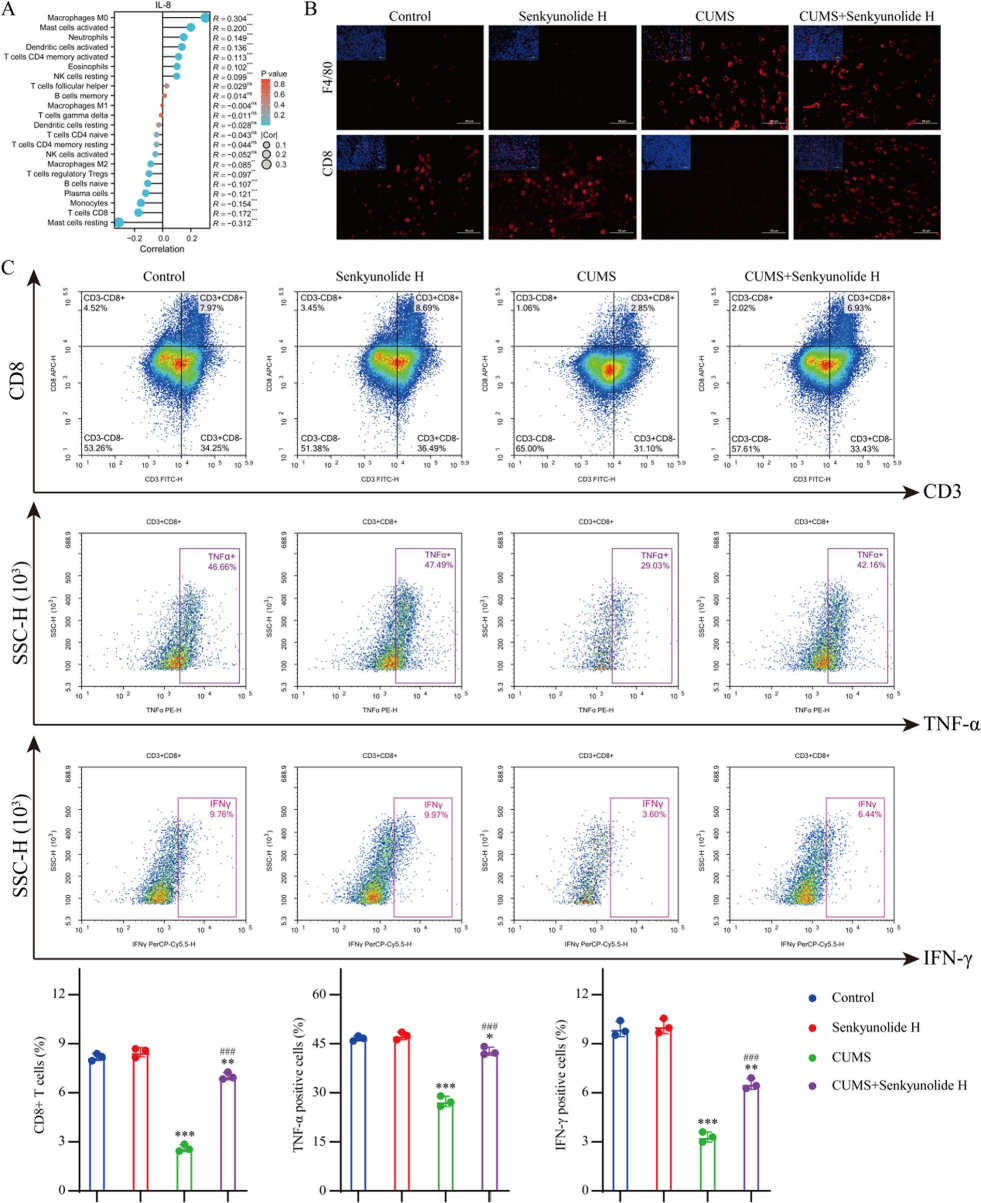
Effect of senkyunolide H on the tumor immune microenvironment. **A** Effect of the IL-8 expression level on immune cell infiltration. **B** IF staining of tumor tissue. **C** Immune infiltration of tumor tissue was analyzed by flow cytometry. The data are expressed as the means ± SDs; ^*^P < 0.05, ^**^P < 0.01, ^***^P < 0.001 in comparison with the CUMS−/senkyunolide H− group; ^###^*P* < 0.001 in comparison with the CUMS+/senkyunolide H− group

The chemokine system is closely related to the immune system. Therefore, we used the TCGA database to analyze the relationship between IL-8 expression levels in breast cancer and the infiltrating components of immune cells in the TME. In invasive breast carcinoma, macrophage infiltration was significantly increased in the tumor immune microenvironment in patients with high IL-8 expression, whereas the infiltration of CD8 + T cells was significantly inhibited (Fig. 6A). Therefore, we examined the composition of the immune microenvironment of each tumor tissue in vivo using IF. IF staining analysis revealed a marked increase in the macrophage count within the tumor tissue of CUMS model mice, whereas a significant reduction in the number of CD8+ T cells was observed. Although senkyunolide H had no significant effect on the number of macrophages, it significantly reversed the decrease in the number of CD8+ T cells in the CUMS model (Fig. 6B). We further verified the cytotoxic function of CD8+ T cells using flow cytometry. These findings indicated that the CUMS model not only led to a reduction in the CD8+ T-cell population within the tumor immune microenvironment but also impaired the antitumor activity of these cells. Although senkyunolide H had no notable effect on CD8+ T cells in the tumor immune microenvironment of nondepressed mice, it significantly increased the number of CD8+ T cells and enhanced their cytotoxic function in the tumor microenvironment of CUMS model mice (Fig. 6C).

## Discussion

In the past, the medical treatment of breast cancer mainly involved targeted therapy and endocrine therapy. However, because the mechanism by which depression promotes breast cancer progression is very unclear, molecular targets suitable for the development of therapeutic drugs for breast cancer under conditions of depression remain insufficient. As an effective technology for discovering key molecular targets, high-throughput sequencing has been widely used in bioscience research. Moreover, in the past, due to the lack of attention given to the emotional state of patients and the exclusive focus on the breast cancer itself, few studies have focused on the biological characteristics of patients with breast cancer and depression.

In this study, using transcriptomic data from clinical samples of breast cancer patients, we demonstrated that IL-8 expression is increased in the breast cancer tissues of depressed patients. IL-8 typically has two receptors, CXCR1 and CXCR2 [23]. By studying these two receptors, we further confirmed that CXCR2 activation can significantly promote breast cancer growth. Previous studies reported that depression promotes breast cancer progression [24], and other reports have shown that the tumor microenvironment of depressed patients has abnormally increased levels of inflammatory factors [25]. However, previous studies have not revealed how depression regulates tumor growth by affecting inflammatory factors. We demonstrated that high IL-8 levels in the tumor microenvironment of depressed patients activated CXCR2, mediating the cancer-promoting effects of depression. Despite the lack of IL-8 in mice, depression can still promote the growth of breast cancer through CXCR2. The reason may be that CXCR2 activation can be affected by not only IL-8 signaling but also CXCL1 signaling [26], indicating that CXCR2 is the key target through which depression promotes the progression of breast cancer. Previous studies and our data also confirmed that depression promotes an increase in CXCL1 levels in the TME [27, 28]. Therefore, targeted intervention with CXCR2 may be more effective against breast cancer associated with depression.

In cancer studies, CXCR2 activation has been shown to promote the activation of tumor proliferation pathways [29]. PI3K/AKT signaling pathway activation by CXCR2 contributes to enhanced cell survival [30]. In addition, the ability of CXCR2 to promote cell survival can also be achieved by activating the Raf/MEK/ERK signaling pathway [31]. We demonstrated that the tumor CXCR2-mediated PI3K/AKT signaling pathway is activated during depression, thus promoting breast cancer cell proliferation. On this basis, we found that senkyunolide H binds to CXCR2, inhibits the activation of CXCR2 by inflammatory factors (such as IL-8 and CXCL1), and blocks the pro-breast cancer effect of depression.

Virtual drug screening using molecular docking is one of the important methods of new drug discovery. Identifying disease-specific targets and then performing high-throughput screening for drugs that can target and interfere with these targets has become the most common method used in the development of drugs for disease treatment. In addition, past drugs tended to focus only on the disease as a whole, with limited consideration for the specific state of the disease. Therefore, the drugs developed under the guidance of this idea easily lead to large adverse drug reactions because the targets of the disease are too broad. Moreover, it is also easy to miss the specific biomarkers that the disease presents in a particular state, leading to the omission of drugs that could otherwise treat the disease. In the field of antitumor drug development, IL-8 and CXCR2 are two important targets. Currently, monoclonal antibodies targeting IL8 (e.g., HuMax-IL8 of Bristol Myers Squibb and ABX-CXCL8 of Abgenix) and receptor inhibitors targeting CXCR2 (e.g., Navarixin of Merck & Co., Inc., AZD5069 of AstraZeneca, and SX-682 of Syntrix Biosystems) have been used to treat advanced tumors; however, clinical studies have shown that the efficacy of these drugs is inconsistent [32–35]. Some trials have shown good antitumor effects, whereas others have shown poor efficacy. This may be because these clinical trials were not designed to focus on the effect that patient mood can have on IL-8 expression levels and thus on the efficacy of the drug. This study revealed that senkyunolide H regulates CXCR2 in the treatment of breast cancer in the context of depression, but it does not have a significant inhibitory effect on tumors not associated with depression. Although senkyunolide H has been reported to have a therapeutic effect on a variety of diseases and has good safety [15, 36, 37], senkyunolide H has never been reported to have a therapeutic effect on cancer. This is largely because, in the past, people only paid attention to the cancer itself and did not pay attention to the impact of patients’ emotions and other states on cancer, thus ignoring the therapeutic effect of senkyunolide H on cancer in a specific state. Senkyunolide H has no inhibitory effect on the breast in the nondepressed state possibly because CXCR2 is not activated in the nondepressed state. This also indicates that the target of CXCR2 is disease-specific and that antitumor therapy based on this target can effectively reduce the side effects of drugs.

Depression not only influences tumor cells but also has an impact on the tumor immune microenvironment [38, 39]. Using bioinformatics analysis, we found that macrophages and CD8+ T cells were most closely associated with IL-8 expression levels in breast cancer. We found that the number of macrophages increased significantly in the tumor immune microenvironment of CUMS mice. Studies have shown that the main source of IL-8 and CXCL1 is macrophages [40], suggesting that depression may promote IL-8 and CXCL1 secretion by inducing macrophage proliferation and activation. In addition, we found that senkyunolide H had no significant effect on macrophages or CXCL1 in the immune microenvironment, which also indicates that the target of the action of senkyunolide H is not associated with inflammatory factor production. CXCR2 is expressed not only on tumor cells but also on T cells. It has been reported that the activation of CXCR2 on T cells can reduce the number of T cells and inhibit their cytotoxic function [41, 42]. Our data revealed that depression significantly reduces the number of CD8+ T cells in the breast cancer tumor immune microenvironment, the antitumor function of T cells is significantly inhibited, and senkyunolide H significantly reverses these changes. Multipathway therapy is beneficial for enhancing the antitumor effect of drugs and reducing the risk of drug resistance. Therefore, senkyunolide H is a very promising candidate for the treatment of breast cancer in the context of depression. This study provides a paradigm for the mechanistic research and drug development of diseases such as breast cancer associated with depression, that is, a focus on the target in a specific state of a disease.

On the basis of the above findings, this study can further enhance the representativeness and clinical significance of the results in the future. For example, in the MR analysis of this study, only the entire breast cancer population was analyzed. To conduct further stratified analysis of the population of patients with breast cancer and depression, the conclusions should be more representative. This approach will also increase the precision of the potential target population for senkyunolide H.

## Conclusions

In this study, we revealed that CXCR2 is a key target that mediates the procancer effect of depression and that senkyunolide H can reverse the probreast cancer effect of depression by regulating the inhibition of CXCR2 activation.

## Data Availability

The datasets used and/or analyzed during the current study are available from the corresponding author upon reasonable request.

## Abbreviations

AKT: Protein kinase B
CD8: Cluster of differentiation 8
CETSA: Cellular thermal shift assay
CUMS: Chronic unpredictable mild stress
CXCL1: C-X-C motif chemokine ligand 1
CXCR1: CXC-chemokine receptor 1
CXCR2: CXC-chemokine receptor 2
DEGs: Differentially expressed genes
F4/80: Mouse EGF-like module-containing mucin-like hormone receptor-like 1
GAPDH: Glyceraldehyde-3-phosphate dehydrogenase
HAMA: Hamilton anxiety scale
HAMD-17: Hamilton depression scale
HPA: Hypothalamic‒pituitary‒adrenal
IF: Immunofluorescence
IL-8: Interleukin-8
Ki-67: Nuclear-associated antigen ki-67
MR: Mendelian randomization
MTT: Methylthiazolyldiphenyl-tetrazolium bromide
NC: Negative control
OCT: Optimal cutting temperature
p-AKT: Phosphorylated AKT
PCNA: Proliferating cell nuclear antigen
PI3K: Phosphatidylinositol 3-kinase
PVDF: Polyvinylidene fluoride
RPKM: Reads per kilobase per million mapped reads
SNPs: Single nucleotide polymorphisms
TAM: Tumor-associated macrophages
TBST: Tris-buffered saline plus Tween-20
TCGA: The Cancer Genome Atlas
TROP2: Trophoblast cell surface antigen-2
WB: Western blotting
WT: Wild type
